# Supplementary material for: Distinct Changes in Calpain and Calpastatin during PNS Myelination and Demyelination in Rodent Models
Source: Int J Mol Sci. 2022 Dec 6;23(23):15443. doi: 10.3390/ijms232315443 (PMC9737575; doi:10.3390/ijms232315443)
Supplement: Supplementary file 1 [file ijms-23-15443-s001.zip › ijms-1724447-supplementary.pdf]

Supplementary Materials

	Shapiro-Wilk Test			Student's t-test	
**Average Number of Nodes**	**W**	***P*-value**	**F test *P*-value**	**t, df**	***P*-value**
Wild-type	0.8521	0.1635	0.2183	t = 11.83<br>df = 9	<0.0001
Trembler-J	0.9583	0.7963	-	-	-

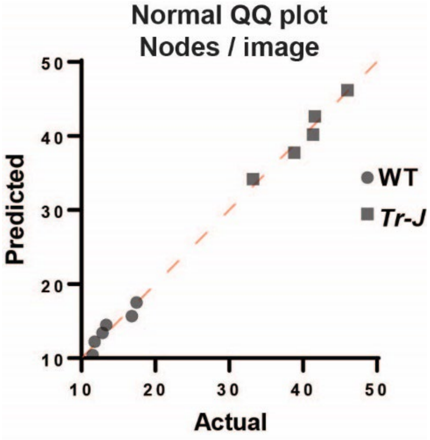

Supplementary S1. Statistical information for Figure 2.

CAPN1	Shapiro-Wilk Test W	*P*-value	F test *P*-value	Student's t-test t, df	*P*-value
Wild-type	0.8623	0.1972	0.4774	t = 2.863<br>df = 9	0.0187
Trembler-J	0.9301	0.5974	-	-	-
**CAPN2**					
Wild-type	0.9627	0.84	0.3585	t = 1.981<br>df = 9	0.0789
Trembler-J	0.8488	0.1908	-	-	-
**Calpastatin**				**Welch's t-test**	**Welch's t-test *P*-value**
Wild-type	0.9134	0.459	0.0129	t = 2.685<br>df = 4.482	0.0487
Trembler-J	0.9218	0.5416	-	-	-
**Calpain Activity Assay**				**Student's t-test**	**Student's t-test *P*-value**
Wild-type	0.9605	0.6179	0.2739	t = 5.629<br>df = 4	0.0049
Trembler-J	0.979	0.7234	-	-	-

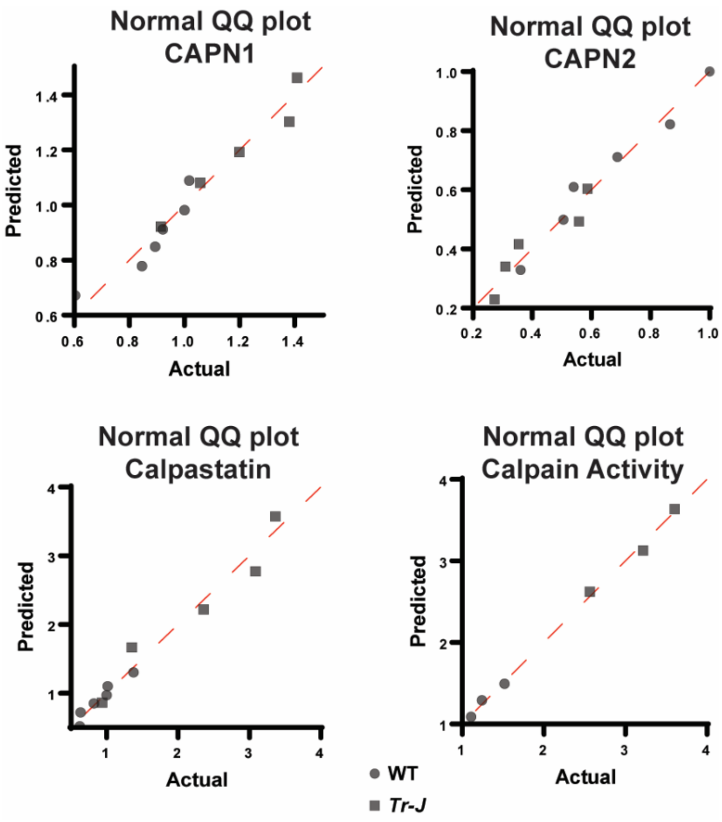

Supplementary S2. Statistical information for Figure 3.

	Shapiro-Wilk Test				
	**W**	***P*–value**	**One Way ANOVA with Tukey Multiple Comparisons**	**Mean Diff.**	**Adjusted *P*-value**
Lysolecithin	0.9219	0.5192	Lysolecithin vs. Vehicle	−2.784	0.0023
Vehicle	0.9527	0.7621	Lysolecithin vs. Intact	−2.593	0.0041
Intact	0.8855	0.2952	Vehicle vs. Intact	0.1918	0.9561

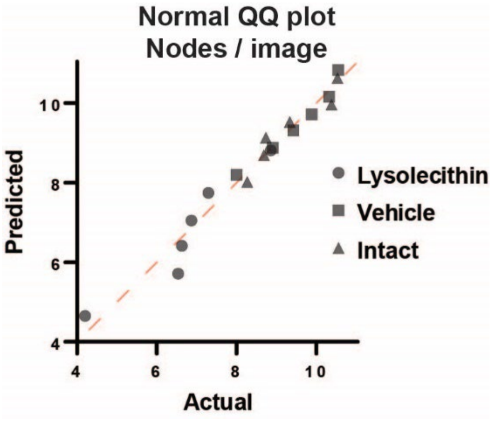

Supplementary S3. Statistical information for Figure 4.

	Shapiro-Wilk Test			Student's t-test	
**CAPN1 80kDa**	**W**	***P*-value**	**F test *P*-value**	**t, df**	***P*-value**
Vehicle	0.8664	0.2123	0.277	t = 2.295<br>df=10	0.0446
Lysolecithin	0.919	0.4982	-	-	-
**CAPN1 76/78 kDa**					
Vehicle	0.9392	0.6531	0.9504	t = 4.117<br>df = 10	0.0021
Lysolecithin	0.8175	0.084	-	-	-
**CAPN2**					
Vehicle	0.9289	0.5714	0.9139	t = 0.9294<br>df = 10	0.3745
Lysolecithin	0.9631	0.843	-	-	-
**Calpastatin**					
Vehicle	0.9133	0.4588	0.4704	t = 5.445<br>df = 10	0.0003
Lysolecithin	0.8547	0.1716	-	-	-
**NF-M**					
Vehicle	0.9613	0.8297	0.224	t = 0.7975<br>df = 10	0.4437
Lysolecithin	0.8255	0.0985	-	-	-
**DRP2**					
Vehicle	0.9293	0.5751	0.8455	t = 12.03<br>df = 10	<0.0001
Lysolecithin	0.8517	0.1625	-	-	-

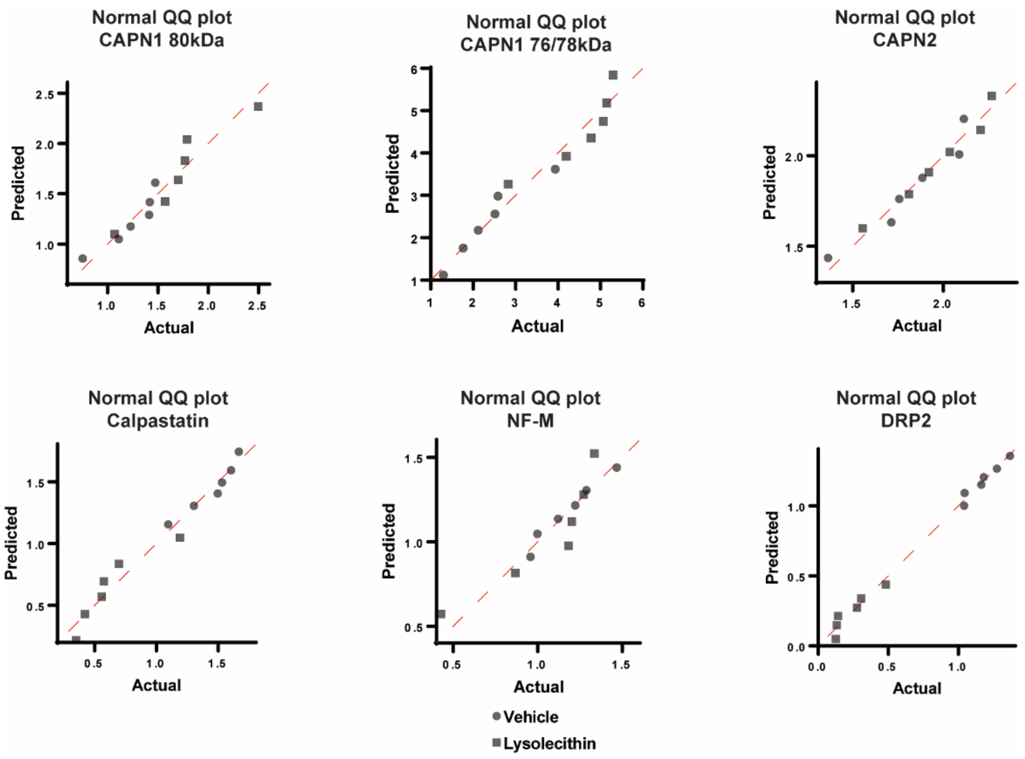

Supplementary S4. Statistical information for Figure 5.

	Shapiro-Wilk Test			Student's t-test	
**CAPN1 80kDa**	**W**	***P*-value**	**F test *P*-value**	**t, df**	***P*-value**
Intact	0.9554	0.7839	0.1267	t = 0.5972<br>df = 10	0.5637
Crush	0.9562	0.7902	-	-	-
**CAPN1 76/78 kDa**					
Intact	0.8424	0.1366	0.4762	t = 4.753<br>df = 10	0.0008
Crush	0.9163	0.479	-	-	-
**CAPN2**					
Intact	0.8214	0.0907	0.622	t = 3.316<br>df = 10	0.0078
Crush	0.8307	0.1091	-	-	-
**Calpastatin**					
Intact	0.9491	0.7331	0.2778	t = 1.497<br>df = 10	0.1654
Crush	0.9361	0.6282	-	-	-
**NF-M**					
Intact	0.9828	0.9645	0.0898	t = 7.082<br>df = 10	<0.0001
Crush	0.9015	0.3829	-	-	-
**DRP2**				**Welch's t-test**	**Welch's t-test *P*-value**
Intact	0.9507	0.7461	0.0119	t = 31.57<br>df = 5.719	<0.0001
Crush	0.9375	0.6392	-	-	-

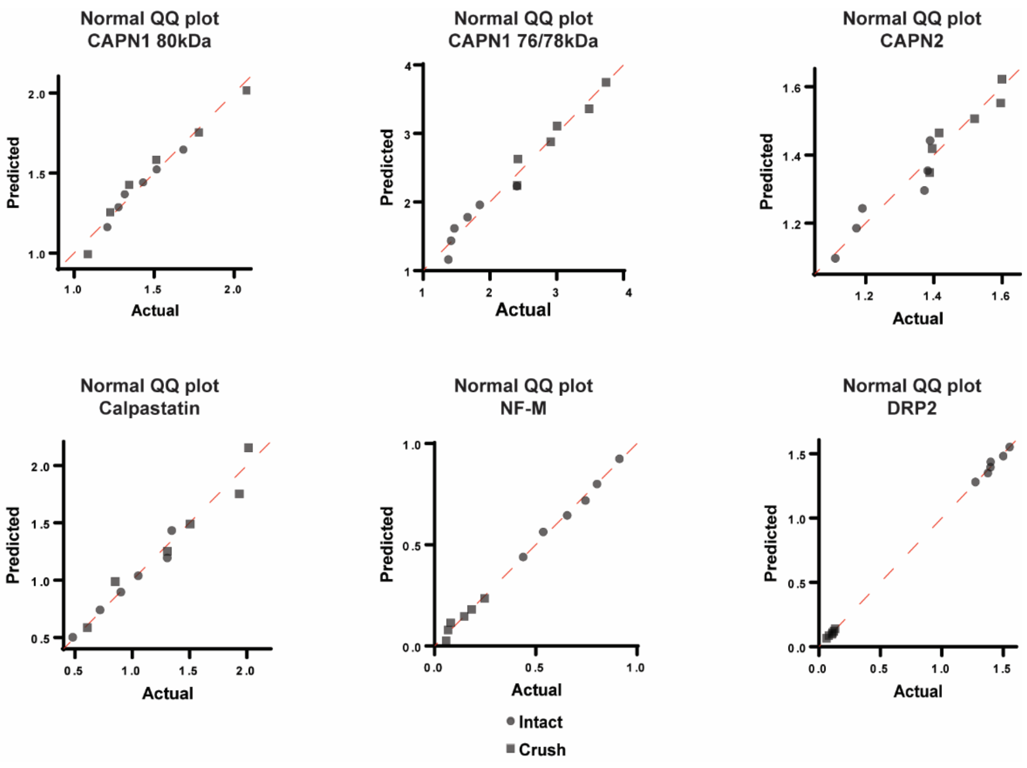

Supplementary S5. Statistical information for Figure 6.
